# Direct Patterning of CsPbBr_3_ Nanocrystals via Electron-Beam Lithography

**DOI:** 10.1021/acsaem.1c03091

**Published:** 2022-01-18

**Authors:** Christian D. Dieleman, Julia van der Burgt, Neha Thakur, Erik C. Garnett, Bruno Ehrler

**Affiliations:** † Center for Nanophotonics, AMOLF, Science Park 104, 1098 XG Amsterdam, The Netherlands; ‡ Advanced Research Center for Nanolithography, Science Park 106, 1098 XG Amsterdam, The Netherlands

**Keywords:** perovskite, nanocrystals, nanopatterning, electron-beam lithography, photoluminescence, AFM, FTIR, nanophotonics

## Abstract

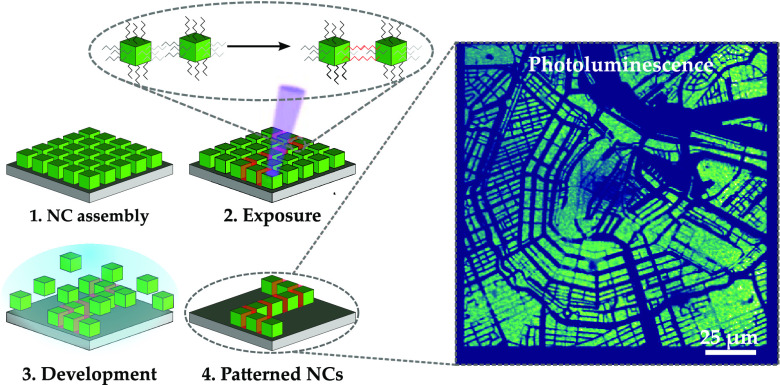

Lead-halide perovskite (LHP) nanocrystals have proven themselves as an interesting material platform due to their easy synthesis and compositional versatility, allowing for a tunable band gap, strong absorption, and high photoluminescence quantum yield (PLQY). This tunability and performance make LHP nanocrystals interesting for optoelectronic applications. Patterning active materials like these is a useful way to expand their tunability and applicability as it may allow more intricate designs that can improve efficiencies or increase functionality. Based on a technique for II–VI quantum dots, here we pattern colloidal LHP nanocrystals using electron-beam lithography (EBL). We create patterns of LHP nanocrystals on the order of 100s of nanometers to several microns and use these patterns to form intricate designs. The patterning mechanism is induced by ligand cross-linking, which binds adjacent nanocrystals together. We find that the luminescent properties are somewhat diminished after exposure, but that the structures are nonetheless still emissive. We believe that this is an interesting step toward patterning LHP nanocrystals at the nanoscale for device fabrication.

## Introduction

Perovskite nanocrystals are an interesting material platform for a diverse range of applications. Their simple colloidal synthesis allows for cheap solution processing and the material has an easily tunable band gap. In other semiconductor colloidal nanocrystals, like II–VI quantum dots, the properties like emission and absorption are most commonly tuned by the quantum confinement effect arising from their small size^1,2^ and to a lesser extent by their composition, as that requires precise alloying.^3,4^ The band gap of perovskite nanocrystals, however, can be readily manipulated via compositional changes, most easily by halide exchange.^5^ Mixing of the halides allows for gradual band-gap adjustments.^5,6^ Moreover, many different precursors and elements form the perovskite crystal structure, allowing easy substitution of elements and a wide library of possibilities in adapting the material to have desired properties. For instance, by replacing cations, one can form methylammonium-based^7^ or formamidinium-based^8^ perovskite nanocrystals, and the often-used lead metal can be replaced to form tin-based perovskites.^9^ Organic ligands, which passivate the surface of the crystals and provide colloidal stability, can also influence the electronic environment of the nanocrystals and alter the shape during synthesis. This allows perovskite nanocrystals to be suitable for many applications including photovoltaics,^10,11^ photodetectors,^12,13^ scintillators,^14,15^ and light-emitting diodes (LEDs).^16−18^ The wide applicability is enabled because perovskites can be efficient emitters, exhibiting long charge-carrier lifetimes and defect resistance. Patterning active materials like these is a useful way to expand their tunability as it may allow more intricate designs that can improve efficiencies or expand the functionality. Patterning materials at the size of the wavelength of light they can emit or absorb can give rise to nanophotonic effects, such as increased absorption,^19^ amplified stimulated emission^20^ or changes in the wavelength,^21^ and the direction of emitted light^19,21^ independent from the original material properties. In previous work, we showed a method to pattern colloidal quantum dots (CQDs) directly by extreme UV lithography (EUVL) or e-beam lithography (EBL) without affecting their luminescent properties significantly.^22^ In this work, we expanded this method to include perovskite nanocrystals and show that these crystals can also be patterned to create structures down to 100s of nanometers that still show luminescence.

Most of the works on patterning perovskite nanocrystals do not make use of direct lithography. Some approaches include (template-assisted) self-assembly,^20,23,24^ laser-assisted deposition,^25,26^ ink-jet printing,^27^ or multistep lithography.^28,29^ Previous work on patterning perovskites via lithography has been carried out by Palazon et al. who showed that the exposure of perovskite nanocrystals to an X-ray photoelectron spectroscopy (XPS) source could impede ion exchange in the crystals and reduce their solubility.^30^ They also showed initial patterning with EBL but not with a detailed analysis of its limits and mechanism. It is not obvious that direct patterning with lithographic techniques is straightforward. Although perovskites are often described as “defect-resistant” due to the mobile nature of ions in the crystal, the material also has stability issues, induced by exposure to oxygen, water, or even long exposure to light.^31^ In scanning electron microscopy (SEM), it is observed that perovskite crystals can be destroyed under the electron beam.^32^ Even though the damage in SEM imaging is induced by the high dose from repetitive scanning across the probe, the highly energetic (but lower-dose) electron beam used in e-beam lithography could also lead to damage of the perovskite nanocrystals, rather than induce local chemical changes that are necessary for direct patterning.

Here, we synthesize CsPbBr_3_ nanocrystals, spin-coat them into thin films, and expose them directly to an electron beam in a commercial lithography machine. Upon exposure and subsequent development of the films in various solvents, we can create well-defined perovskite structures on the substrate. Feature sizes can be as small as 100s of nanometers. We find that the chemical changes in the ligands induce a cross-linking reaction as well as partial detachment of the ligands that creates a less soluble assembly of nanocrystals. As the required doses are relatively high, we do find some damage to the material in terms of a reduced photoluminescence quantum yield (PLQY). The developed samples are, however, still luminescent, and we believe that this technique is suitable for device fabrication, especially when combined with more efficient cross-linking ligands or repassivation of the crystal surface after processing.

## Results and Discussion

**Figure 1:**
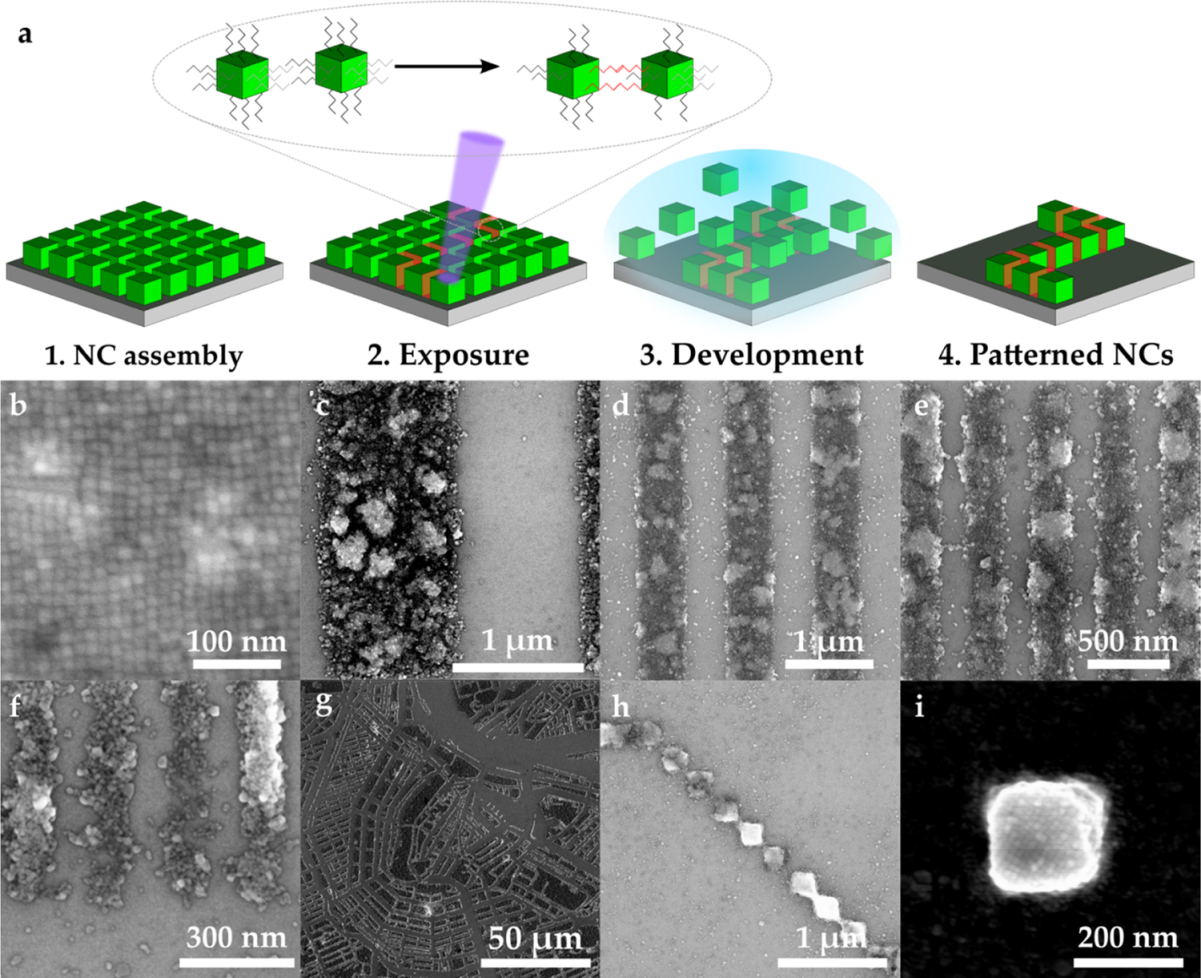
(a) Processing of the patterned nanocrystals. After spin coating (1), the cubic crystals are exposed to a high-energy electron beam (2), which induces cross-linking between ligands of adjacent nanocrystals, as well as some loss of ligands (indicated by the orange layer between the crystals). This makes the new clusters less soluble during the development phase (3), allowing for the selective dissolution of nonexposed nanocrystals and leaving intended structures of nanocrystals behind on the substrate (4). SEM images of unpatterned (b) and e-beam-patterned (c–i) CsPbBr_3_ nanocrystals at a dose of 2000 μC cm^–2^. Panel (b) shows the crystals after synthesis, spin-coated into thin films. Panels (c–f) show lines of nanocrystals of 1 μm, 500 nm, 200 nm, and 100 nm widths, respectively. Thinner lines exhibit more apparent line-edge roughness as small variations in particle size have a larger effect on the relative smoothness of the line edge. (g) A pixelated image of the map of Amsterdam consisting of 200 nm × 200 nm pixels. (h, i) Detailed images of these pixels. These structures can be placed with high precision and touch in the corners.

We chose to synthesize and pattern CsPbBr_3_ nanocrystals as they are known to be relatively stable and have a high PLQY, especially compared to iodide-containing compounds.^8,33^ We synthesize CsPbBr_3_ nanocrystals by adapting the approach of Protesescu^5^ and Lu,^34^ which comprises the hot injection of a Cs-oleate into a solution of PbBr_2_, oleylamine (OlAm), and oleic acid (OA) in 1-octadecene. After synthesis and purification, we are left with cubic CsPbBr_3_ nanocrystals of around 15 nm in toluene. These crystals can be spin-coated onto silicon substrates to form thin layers of nanocrystals with an average thickness of 100 nm. A typical film of nanocrystals can be seen in Figure 1b. The cubic particles arrange themselves into larger-ordered structures. These films are then directly exposed to the electron beam of a commercial EBL machine (Raith Voyager). This machine operates at 50 keV, and different beam currents can be used for exposing the perovskite films. We expose the films to doses between 100 and 10 000 μC cm^–2^ in line patterns with a width of 50, 100, 200, and 500 nm as well as 1, 2, and 5 μm. After the exposure, we develop the samples in a solvent mixture of chloroform and refrigerated tetrahydrofuran (THF), after which only regions exposed to the electron beam remain on the substrate, resulting in the desired structures formed by perovskite nanocrystals. The schematic of this process can be seen in Figure 1a. Some of these structures can be observed in Figure 1b–h. This behavior, where material becomes partially insoluble upon electron-beam exposure, is reminiscent of negative photoresists that are cross-linked by the electron beam. Unlike commercial photoresists that are typically converted with doses on the order of 100s of μC cm^–2^ due to their high density of functional, cross-linkable groups, the relatively large perovskite nanocrystals need a higher exposure dose of 2000 μC cm^–2^ to obtain a clear structure. This makes the current system less suitable for high-volume manufacturing; however, we have also observed initial results that it is possible to use EUVL in a similar fashion to pattern these materials (Figure S1). With optimization of this method, the process can become viable for high-volume manufacturing in the future. Figure 1c–f shows examples of different line patterns formed by the e-beam patterning. One can observe lines of 1 μm, 500 nm, 200 nm, and 100 nm, respectively. In all cases, the individual nanocrystals can be observed. Down to 500 nm, the lines are very well defined, with remarkably straight edges. When scaling down further, small imperfections start to influence the line-edge roughness to a greater extent. As the nanocrystals we use are cubic, good stacking and self-assembly are beneficial for straight edges. Another factor playing a role in the line-edge roughness is the particle size distribution. Even though in general the nanocrystals are of similar size and shape, larger particles tend to be harder to redissolve during the development process and can lead to less optimal particle assembly. This may disrupt the line edges as is visible in the thinner lines of 200 and 100 nm in Figure 1e,f.

A balance between underexposure and overexposure of the features exists. When underexposing, insufficient material remains on the substrate to form the desired features, while overexposure causes features to blur and leads to more particles remaining on the substrate where not intended. Some examples of this behavior can be found in Figure S2 of the Supporting Information. The optimum doses are relatively high as compared to those of commercial resist materials as well as our previous work with CdSe and PbS quantum dots,^22^ on the order of 2000 μC cm^–2^. This may be related to the larger size of the perovskite crystals used in this work in contrast to the crystal size of previous systems. As the nanocrystal size is growing, the surface-to-volume ratio reduces, and if patterning is dependent on ligand interactions, the reduction of the ratio of ligands to particle volume may be detrimental for the sensitivity. A second reason for the high doses may lie in the binding strength of the ligands to the crystals. Due to the ionic interaction between the ligands and the crystal, ligands on perovskite nanocrystals do not bind as strongly as the ligands in II–VI quantum dots.^35^ This may mean that in order for the patterning mechanism to work, more ligands need to cross-link, as otherwise, during development, some cross-linked ligands may simply release from the surface and do not contribute to the cohesion of the crystals.

Figure 1g shows the map of Amsterdam as a pixelated image. Individual pixels are 200 nm × 200 nm, with an overall image size of 150 μm × 150 μm, making this a map with scaling 1: 23 million. In Figure 1h,i, magnifications of the image can be seen in the form of a line of pixels touching at the corners and a single isolated pixel, respectively. These examples clearly show a resolution of 200 nm, and placement of these structures is accurate at the defined positions. In the single pixel, one can also observe the individual nanocrystals. Here, again the influence of the arrangement of the crystals can be observed as the crystals seem to be oriented at a 45° angle with respect to the sides of the square, leading to slightly truncated corners. Although not the focus of this work, it could be interesting to further optimize the stacking of nanocrystals in the future to match the final orientation of the desired features.

**Figure 2:**
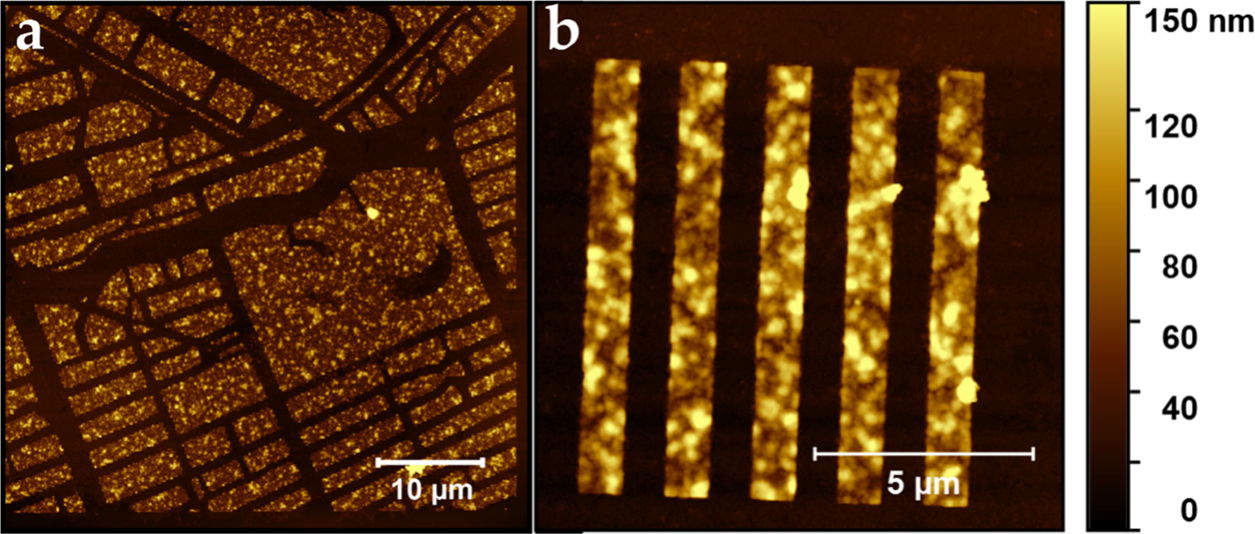
AFM images of patterned CsPbBr_3_ nanocrystals, with a detail of the map of Amsterdam (a) and the 1 μm lines (b). There is good contrast between the intended structures and the substrate. The thickness is around 100 nm.

Figure 2 shows atomic force microscopy (AFM) images of the patterned nanocrystals. Good contrast of the patterns is achieved between the substrate and thick structures on the order of 100 nm. This thickness corresponds to film thicknesses that are relevant for device fabrication. There appears to be an increased roughness in the film after patterning, most likely due to changes in the ligand arrangement after the cross-linking that can cause the local expansion or contraction of the material, possibly reducing the adhesion between two layers of nanocrystals. The development process can also influence roughness, as during the development phase, some crystal facets that are less well protected by ligands locally dissolve and possibly reprecipitate.

**Figure 3:**
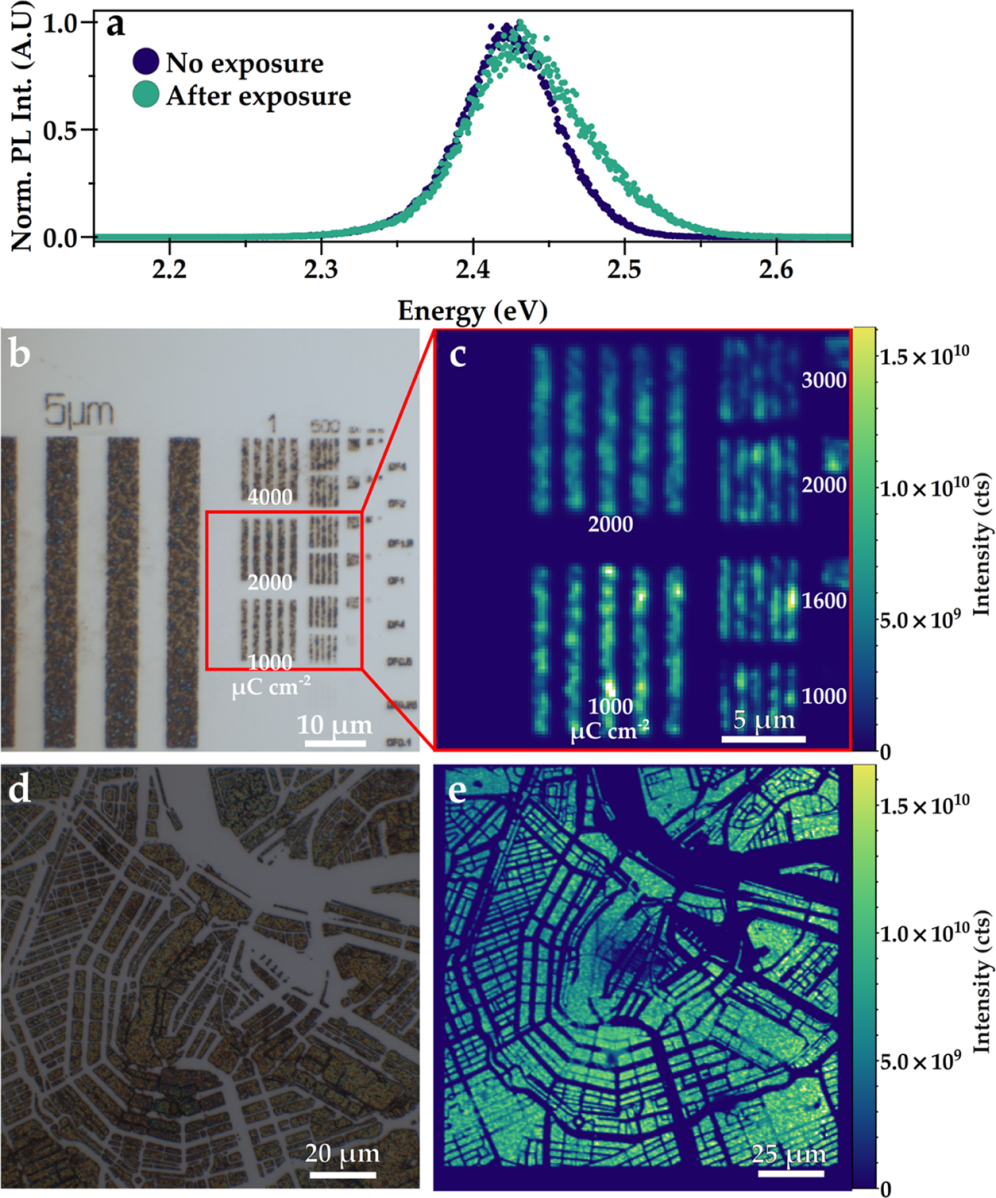
(a) Observed spectrum of patterned and unpatterned CsPbBr_3_ nanocrystals. The spectra are very similar in shape and have a peak emission at 2.42 eV/513 nm. Optical microscope (b, d) and integrated PL intensity maps (c, e) of patterned CsPbBr_3_ nanocrystals. Structures are excited with a 405 nm laser, and PL around the peak emission of the perovskite is integrated. Lines with a width down to 500 nm can be spatially resolved with the optical microscope. An e-beam exposure dose of at least 1000 μC cm^–2^ is needed for good contrast. Doses over 2000 μC cm^–2^ diminish the PL intensity. Exposure doses are shown in the images (b, c). The Amsterdam map is exposed at 2000 μC cm^–2^.

Patterning perovskite nanocrystals does not serve a function if their intrinsic functionality is lost. The photoluminescence (PL) should therefore remain after processing. We spatially map the PL with a confocal microscope system (WITec alpha300 SR), where the structures are excited with a 405 nm laser coupled into the objective. Figure 3a shows the normalized PL spectrum of the CsPbBr_3_ nanocrystals before and after exposure (raw data in Figure S3). We observe the typical emission profile of CsPbBr_3_ with peak emission around 2.42 eV or 513 nm, as observed by others.^5,34^ We see a small broadening at the high-energy side after exposure and an increase in full width at half maximum (FWHM) from 0.070 to 0.085 eV. A general broadening of the PL is expected from the exposure as damaging of the material may create more states from where emission is possible. Figure 3 shows optical microscope and integrated PL intensity maps side-by-side for line patterns (b, c) and the Amsterdam map (d, e). The exposed and developed structures are still very brightly luminescent and show the same emission line shape as before exposure. The line patterns visible in the PL map have a width of 1 μm in the left column, 500 nm in the middle column, and 200 nm in the rightmost column. Down to 500 nm, lines can be resolved by the microscope due to the high NA of the objective (0.9). The 200 nm lines cannot be spatially resolved, even though emission is detectable from this block of lines. The exposure doses used for patterning in Figure 3 are 2000 and 1000 μC cm^–2^ for the 1 μm lines and 3000, 2000, 1600, and 1000 μC cm^–2^ for the 500 nm lines from top to bottom, as shown in the figure. All lines in this region are similarly resolved after patterning, but PL intensity is slightly less bright for higher doses. For the 200 nm lines, which are written with the same exposure doses as the 500 nm lines, the story is slightly different. In this case, the lower dose of 1000 μC cm^–2^ is not enough to form a thick structure and thus PL is not very bright. At least 1600 μC cm^–2^ is necessary to resolve a clear structure, but both contrast and PL are higher at 2000 μC cm^–2^. At higher doses, the PL decreases again. This raises the question to what extent the patterning and exposure dose affects the PLQY, as the dose should be high enough to resolve the structures but low enough to avoid damage to the semiconductor properties.

**Figure 4:**
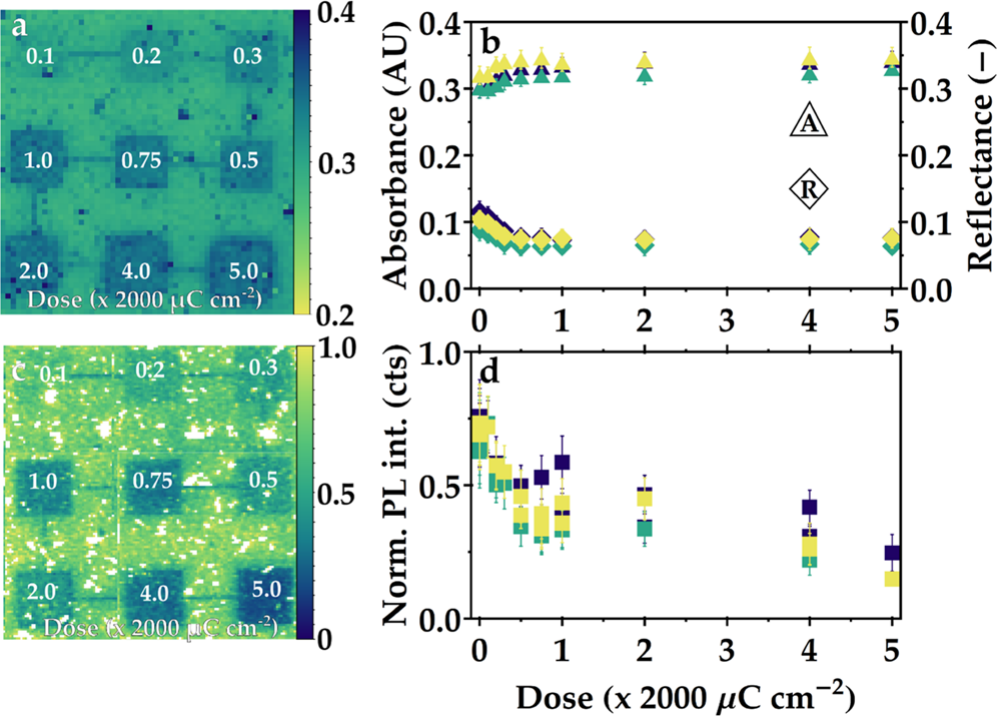
Absorbance, reflectance, and integrated PL intensity of exposed but undeveloped CsPbBr_3_ films. (a) Absorbance map of the exposed nanocrystal film. White labels indicate the dose factor, which should be multiplied by 2000 μC cm^–2^. Individual exposure fields are 50 μm × 50 μm. (b) Absorbance (triangles) and reflectance (diamonds) as a function of the exposure dose of three different samples. (c) PL intensity map with the relative PL intensity per exposure dose. (d) Average PL intensity as a function of exposure dose for three different samples.

To estimate the changes in PLQY, we expose a film of CsPbBr_3_ nanocrystals to different e-beam doses without development and spatially map the absorbance as well as PL. Measuring absorbance requires a transparent substrate. As glass or quartz is not conductive, however, patterning a perovskite layer on top of glass is not possible. The exposure would cause charging of the sample and drifting of the electron beam, which makes proper patterning impossible. Therefore, indium tin oxide (ITO)-coated glass substrates were used to allow for dissipation of charges under exposure. We expose the sample to different doses ranging from 200 to 10 000 μC cm^–2^ (corresponding to dose factor 0.1–5.0). Subsequently, the absorbance and reflectance at 405 nm, the same wavelength used for excitation for the PL measurement, of the exposed area were mapped in an integrating sphere setup described in earlier work.^36^ This setup allows us to record the absorption and reflection at the same time. Inspection under the optical microscope already shows that exposure leads to changes in absorbance, as areas exposed to higher doses appear darker (see Figure S4). Figure 4a shows an absorbance map of exposure fields on an undeveloped CsPbBr_3_ film. Figure 4b shows the measured absorbance (triangles) and reflectance (diamonds) as a function of exposure dose. The error bars indicate the standard deviation of the measured absorbance over the pixels of each dose in the exposure field. We show the results of three samples, which show a similar trend, although absolute absorbance and reflectance values may change from sample to sample. The full absorbance and reflection maps can be found in Figure S5 in the Supporting Information (SI). We observe an increase of the absorbance from 0.30 ± 0.02 of the unexposed film to about 0.34 ± 0.01 at 2000 μC cm^–2^, after which the absorption only increases very slightly even when the exposure dose is 5 times higher. This increase in absorption may be an explanation for the observed broadening of the PL on the high-energy side of the spectrum (Figure 3a). It is not immediately obvious why the absorption increases. One explanation could be an increased film thickness. In AFM, however, we do not observe any clear increase in film thickness (SI, Figure S6). Another explanation could be the outgassing of volatile organic compounds from the film, which might originate from decomposition products of the organic ligands. The relative decrease in the organic content of the film could give rise to absorption changes. In this case, however, one would expect a decrease in film thickness and a strong reduction in IR absorption of organic functional groups. As shown in an upcoming section, this is not the case. A change in the refractive index of the material, due to changes in the electronic environment or different packing of the crystals, is also a possibility, which would change the reflectance and absorption. From Figure 4b, we also observe a change in reflectance as a function of dose, with a similar but opposite trend to the absorption, as it decreases from 0.12 ± 0.02 to 0.07 ± 0.01. A change in refractive index is therefore a reasonable hypothesis. To determine what change in refractive index could induce changes in absorption and reflection like those observed, we perform simple optical modeling of our system based on a transfer matrix calculation model by the McGehee group,^37,38^ in turn based on the work of Pettersson et al. and Peumans et al.^39,40^ The results can be found in Figure S7 of the Supporting Information. We focus our analysis on a single wavelength (the excitation wavelength λ = 405 nm), as we expect the changes to be qualitatively similar for the full spectrum. We find that a reduction in the refractive index *n* from 1.9 to around 1.75 may result in similar changes as observed in our measurements and is therefore the likely origin of the observed changes in optical properties. These changes could result from the cross-linking and therefore rearrangement of the ligands (see below) although understanding the exact origin of the change in refractive index itself would require further investigation, which is challenging due to the small area of exposure.

Figure 4c shows a typical integrated PL intensity map of one of the exposed samples. The intensity is normalized to the 95th percentile intensity value as some pixels were extremely bright. The mean values and spread of the PL in all of the exposure fields can be found in Figure 4d. We observe a similar but opposite trend to the absorption: an initial fast decrease up to an exposure dose of 2000 μC cm^–2^, from 0.70 ± 0.13 to 0.40 ± 0.07, followed by a plateau and eventually further decrease. The exposure does therefore alter the emission intensity. Most of this change seems to occur up to 2000 μC cm^–2^, which coincides with the dose that yields well-defined features. Higher doses did not change the resolved features significantly, but it does seem to have a further effect on the PL properties.

From the relative changes in absorbance, +26.1%, and PL, −42.9%, one might estimate that the PLQY does decrease quite significantly by a factor of 2. This does, however, assume that the outcoupling is not changed in the exposed areas as the light is collected in an objective above the sample and the emission is not collected in every direction. A change in film roughness might, however, scatter more light into more shallow angles and the potential change in refractive index discussed above might also alter the outcoupling efficiency.

**Figure 5:**
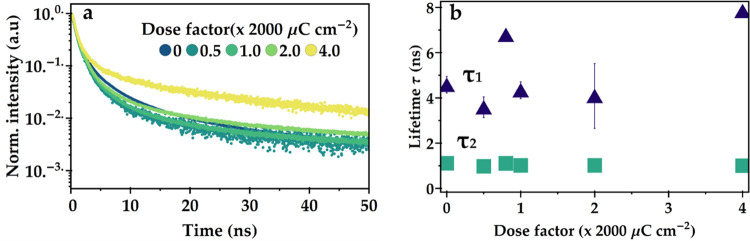
(a) Time-resolved photoluminescence of exposed CsPbBr_3_. (b) Fitted lifetime components.

We measure radiative lifetime to understand the changes we measured in PL intensity. We measure unexposed as well as patterned CsPbBr_3_ films via time-correlated single photon counting (TCSPC) with a pulsed 485 nm excitation laser. We fit the decay curves with a two-exponential model (eq 1), where the first term is a stretched exponential term to take into account the nature of nanocrystals.^22,41^ As they are an assembly of very small, single, isolated crystals, there is likely a distribution of radiative decay rates as these rates can slightly vary from crystal to crystal, more so than in a three-dimensional (3D) bulk material. The stretch factor β is an indicator of how wide the distribution of rates is and can take a positive value below or equal to 1. A β of 1 indicates the limit where only one rate is present, and we can ignore the stretched exponent
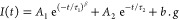
1. where *I*(*t*) is the number of PL counts at time *t*, *A*_n_ is a dimensionless prefactor for each exponential term, τ_n_ is the characteristic lifetime, β is a dimensionless stretched exponential factor, and b.g. accounts for the background counts. The PL decay curves can be found in Figure 5. We find two lifetime components, one around 1 ns and one around 4 ns. The fast decay component is constant over different doses, while the longer component shows more spread. The observed spread is, however, also present from measuring different spots on the same sample. Fitted lifetime components of similar, unexposed samples can be found in appendix Figure S8. The stretched exponential β is stable around 0.75 (appendix Figure S9), which indicates a relatively small distribution of rates. Previously, we assigned the slower component to be the radiative lifetime, while the faster component is related to nonradiative processes. From these data points, however, it is not obvious that there is an increase in the nonradiative processes as the shorter lifetime component does not show a clear trend. It seems that the slower component increases with higher exposure doses, over 4000 μC cm^–2^. This could be an indication that more trapping and detrapping are involved during the emission, causing more delayed fluorescence.^42^

The optical measurements therefore show an overall reduction in PL intensity with increasing exposure dose, although there is still relatively bright luminescence in developed structures with an unchanged line shape. In a similar trend, we observe an increase in absorption, likely due to changes in the refractive index caused by changes in the electronic environment of the crystals. The measured radiative lifetime does not change dramatically with dose, however. Although there are many processes that could influence the measured lifetime, it is possible that the fitted lifetime component is not purely radiative or nonradiative and therefore changes in nonradiative processes are less easily observed or that part of the changes in the measured PLQY result from differences in outcoupling, as speculated above.

**Figure 6:**
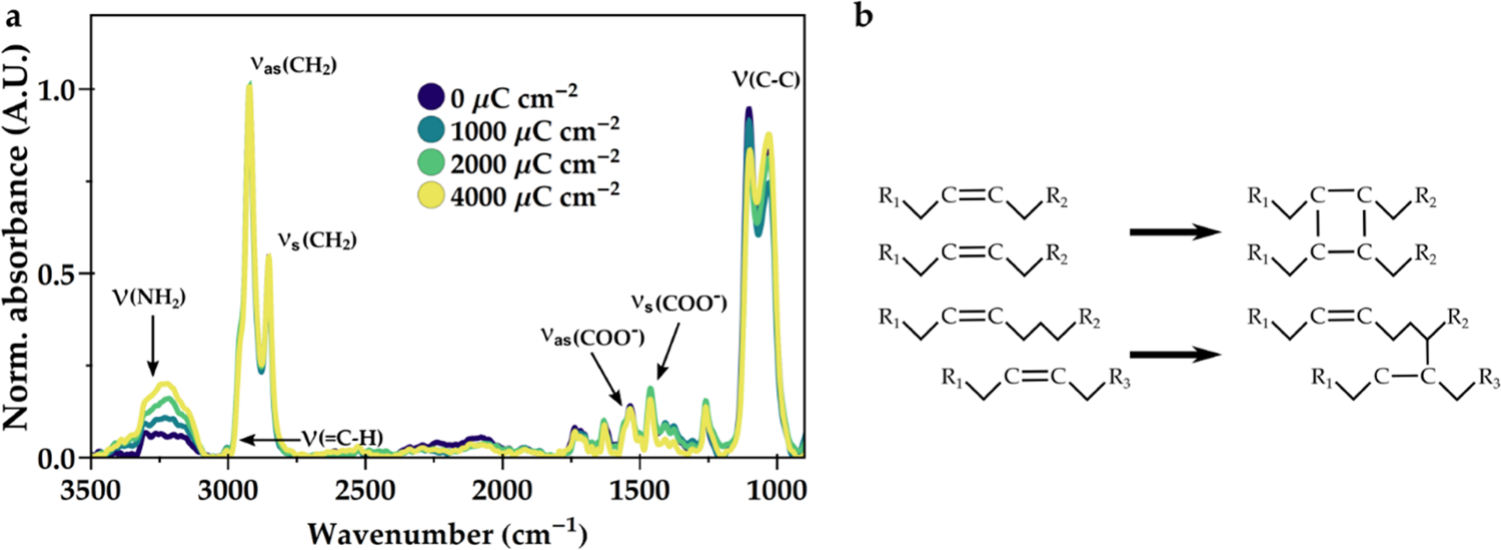
(a) FTIR spectra of e-beam-exposed CsPbBr_3_. The films were exposed to different doses of 50 kV electrons. The spectra are normalized to the peak at 2950 cm^–1^. The main changes are observed in the amine groups and the arrangement of the C–C bonds within the long ligand chains. (b) We propose that the double bonds that are present in both oleic acid and oleylamine can be attacked by radical species upon electron-beam exposure and can cross-link to form new C–C bonds between chains, thereby creating a network of interconnected ligands.

To shed more light on the underlying patterning mechanism, we investigate the changes in the organic part of the perovskite by measuring Fourier transform infrared spectroscopy (FTIR). The spectra of differently exposed perovskite films can be found in Figure 6a. The changes are only small, indicating no major loss of organic material. This observation excludes outgassing of volatile organic compounds as a mechanism for changing absorption. The bands around 2950 and 2850 cm^–1^ correspond to asymmetric and symmetric CH_2_ stretches, respectively. These are the most prominent bands due to the long carbon chains present in both oleic acid and oleylamine. The features around 1450 and 1550 cm^–1^ can be attributed to the COO^–^ anchoring group of oleic acid. These peaks are mostly unaffected by the exposure, indicating a good connection with the inorganic cations in the crystal cores, most likely Cs. An obvious change is the increase in the features between 3100 and 3300 cm^–1^, which can be ascribed to several NH_2_ modes. This change shows that with higher doses, more free amine groups are present in the film. These must originate from the amine binding group, which shows that some oleylamine ligands have detached. This ligand loss could be an explanation for the decrease in PLQY and may have an influence on the long-term stability of the perovskite, as not all facets of the perovskite will be properly passivated. We also observe a change in the wavenumber range around 1000–1100 cm^–1^. These peaks are related to stretches between two carbon atoms in a chain. The ratio between the high-wavenumber peak and the low-wavenumber peak is continuously shifting toward the latter with increasing dose. This points to the creation of new C–C bonds. Overall, we hypothesize that the long ligands, oleylamine and oleic acid, partly cross-link upon exposure, as observed before.^22^Figure 6b shows possible cross-linking pathways. The high energy of the incoming electrons can create free electrons with a broad range of energies. Due to subsequent elastic and inelastic scattering, excited states within the molecule, as well as reactive holes, ionizations, and radicals, are generated.^43^ As the energy of bonds like C–C and C–H are on the order of 3.6 and 4.4 eV, it is possible to break these bonds. Finally, it is possible to capture slower electrons in antibonding orbitals, which will lead to dissociation of bonds and reactive radicals as well.^44^ These reactive species can attack double bonds and promote the formation of new bonds.^45^ The increase in the feature between 1000 and 1100 cm^–1^ shows that new C–C bonds are being created, presumably by cross-linking at the double-bond position. The low concentration of the double-bond feature makes it, however, difficult to properly quantify. In combination with partial ligand detachment, which leads to charged surfaces, the nanocrystals are likely to form larger clusters of interconnected nanocrystals. This clustering strongly reduces the solubility of the crystals, thereby creating an insoluble material. The oleates are not affected, thereby keeping luminescence mostly intact. In e-beam lithography, the high-energy electrons will mostly pass through the patterning material entirely, as can be deduced from Monte Carlo simulations of electron (SI, Figure S10), even with thicker films. Since the patterning mechanism is induced by ligand changes, we believe that the ligands will have a bigger influence on the patterning sensitivity than the core crystals and therefore other perovskite compounds will likely behave in a similar way to CsPbBr_3_. To increase sensitivity, however, other ligand chemistries may be favorable.

## Outlook and Conclusions

We have patterned CsPbBr_3_ nanocrystals in a simple one-step e-beam lithography process into structures down to 100s of nanometer with doses of around 2000 μC cm^–2^. The patterns are well resolved down to about 100 nm and show excellent contrast. We observe a reduction in the relative PLQY of the material after processing, but luminescence remains bright and the radiative decay time scale is relatively stable. We attribute the patterning mechanism to cross-linking of the surface ligands. The ligands, next to passivating the surface and providing colloidal stability, thus also function as the scaffold that insolubilizes the perovskite nanocrystal clusters to form a pattern. The amine ligands, however, partially detach from the crystal, thereby reducing the passivation.

The required dose for patterning is relatively high, and further optimizing the cross-linking chemistry would be valuable for large-scale implementation, especially because this patterning technique is compatible with EUVL. EUV is rapidly becoming the new standard for high-volume lithography. Optimizing the cross-linking by increasing the number of double bonds and using strong anchoring groups should enhance the sensitivity and simultaneously increase stability.

The e-beam patterning of perovskite nanocrystals at the nanoscale presented here can be useful to pursue better-performing devices, for instance, by making use of nanophotonic structures to engineer the emissive properties.

## Experimental Methods

### Chemicals

Hexane (anhydrous) and toluene (anhydrous) were purchased from VWR. Lead bromide (PbBr_2_, 98% pure) was purchased from TCI. Cesium carbonate (Cs_2_CO_3_, 99.5% pure), 1-octadecene (ODE, technical grade 90%), oleic acid (OA, technical grade 90%), oleylamine (OlAm, technical grade 90%), tetrahydrofuran (THF, anhydrous, >99.9% pure), acetone (ACE, analytical grade), isopropanol (IPA, analytical grade), and methanol (MeOH, analytical grade) were purchased from Sigma-Aldrich. Double-side polished silicon wafers were purchased from Siegert Wafer. ITO-coated glass microscopic slides (19 mm × 19 mm) were purchased from Diamond Coatings.

### Nanocrystal Synthesis

Synthesis of the CsPbBr_3_ nanocrystals was performed via a recipe adapted from Lu et al.^34^ and Protesescu et al.^5^ First, cesium oleate precursor was prepared by adding 1.63 g of Cs_2_CO_3_ with 15.8 mL of OA with 34.2 mL of ODE into a three-neck flask in a glovebox to ensure oxygen- and water-free conditions. The flask was attached to a Schlenk line and evacuated 3× and under a flow of N_2_; then, the solution was heated to 110 °C under continuous stirring for 3 h until all precursors were dissolved and a clear solution with the color of melted butter had formed. The solution was cooled to room temperature, and small amounts were taken from this stock solution.

For the final nanocrystal synthesis, 0.138 g PbBr_2_ was combined in a glovebox with 0.5 mL OlAm, 0.5 mL OA, and 10 mL ODE in a 50 mL three-neck flask. The flask was evacuated 3× and refilled with N_2_. Subsequently, the flask was heated to 120 °C and left for 1 h to degas and boil off any water. The precursor dissolved, and a clear, cream-colored solution formed. Subsequently, the temperature was increased to 150 °C and 1 mL of Cs-oleate precursor solution was injected. After a couple of seconds, the solution turned bright yellow/green and the reaction was subsequently quenched in an ice bath. The reaction solution showed bright green fluorescence under UV.

To purify the reaction product, 1 mL of the reaction liquid was mixed with 1 mL of toluene, followed by centrifugation at 12 000 rpm for 10 min. The supernatant was discarded, and the precipitate was dispersed in 200 μL of hexane. The solution was centrifuged for 10 min at 12 000 rpm again, and the supernatant was again discarded. Finally, the precipitate was dispersed in 100 μL toluene and centrifuged 5 min at 4000 rpm. The supernatant was kept as the final solution. This process was repeated until all of the reaction solution was purified.

### Film Preparation

Samples for exposure were made by spin-coating thin films of nanocrystals on clean silicon substrates. The substrates were cleaned by subsequent sonication in soap water, distilled (DI) water, ACE, and IPA and finally 15 min of oxygen plasma. Nanocrystal solutions were filtered (0.2 μm poly(tetrafluoroethylene) (PTFE) filter) and spin-coated at 2500 rpm for 20 s, resulting in films with a thickness of around 100 nm.

### High-Energy e-Beam Exposure

E-beam exposure was done in a Raith Voyager commercial e-beam lithography system with a voltage of 50 kV, an LC30 column mode with a 0.135 nA beam current. Films were developed in a (1:1 vol) mixture of THF and chloroform immediately after exposure.

### Scanning Electron Microscope

SEM images were taken by an FEI Verios 460 at voltages between 5 and 10 kV at 100 pA.

### Atomic Force Microscopy

AFM images were taken on a Veeco Dimension 3100 (Bruker) in tapping mode. Linescan frequency was 1 Hz.

### Photoluminescence

Photoluminescence of samples was measured using a WITec alpha300 SR confocal microscope with a 100× Zeiss objective (NA 0.9). A 405 nm Thorlabs S1FC405 fiber-coupled laser diode was used as an excitation source. A 405 nm notch filter was used to remove the laser light in the detection path, which was coupled to the detector. Light is collected in reflection on a UHTC 300 VIS WITec spectrometer. The PL spectra were converted to the energy scale using a Jacobian transformation.^46^ The setup can be used to record single spectra as well as perform spatial PL mapping.

### Photoluminescence Lifetime Measurements

Fluorescence lifetimes were recorded with a homebuilt TCSPC setup (PicoQuant PDL 828 “Sepia II” and a PicoQuant HydraHarp 400 multichannel) in an inverted microscope with an Olympus 60x Plan Apochromat water immersion objective. The samples were excited by a 485 nm laser (PicoQuant LDH-D-C-485), which was pulsed at a repetition rate of 10 MHz. The excitation laser signal was blocked from the detection path by a Thorlabs FEL-500 long-pass filter in combination with a 488-NF notch filter.

### Absorption Measurements

Absorption measurements were performed in a custom-built integrating sphere setup, described in previous work.^36^ Absorbance was measured at 405 nm by recording the transmission, scattering, and reflection of a 405 nm Thorlabs S1FC405 fiber-coupled laser diode.

### Optical Microscope

Optical microscopic images were recorded with a Zeiss, AxioCam ICc 5 equipped with a 20×/0.2 objective.

## Supporting Information Available

Extra SEM images of EUV and e-beam exposed nanocrystal films, raw PL data, optical microscopic images, full absorption and reflectance maps of exposed nanocrystal films, extra AFM height map, transfer matrix optical modeling of the perovskite film, extra TCSPC data, and Monte Carlo simulations of electron trajectories in CASINO (PDF)
