# Supplementary material for: Direct Patterning of CsPbBr3 Nanocrystals via Electron-Beam Lithography
Source: ACS Appl Energy Mater. 2022 Jan 18;5(2):1672–80. doi: 10.1021/acsaem.1c03091 (PMC8889902; doi:10.1021/acsaem.1c03091)
Supplement: Supplementary file 1 — ae1c03091_si_001.pdf [file ae1c03091_si_001.pdf]

Supporting Information

Direct patterning of $CsPbBr_3$ nanocrystals *via* electron-beam lithography

Christian D. Dieleman[1,2], Julia van der Burgt[1], Neha Thakur[2], Erik C. Garnett[1], Bruno Ehrler[1]

[1]Center for Nanophotonics, AMOLF, Science Park 104, 1098XG, Amsterdam, The Netherlands

[2]Advanced Research Center for Nanolithography, Science Park 106, 1098 XG, Amsterdam, The Netherlands

Corresponding Author:
Bruno Ehrler – ehler@amolf.nl

S1. EUV exposed and developed $CsPbBr_3$ nanocrystals

We exposed some samples to extreme UV light (13.5 nm, 92 eV) in the Swiss Light Source of the Paul Scherrer Institute in Villigen, Switzerland. Using interference lithography[1] we wrote lines with 100 nm pitch. Although we were not able to optimize the system for this type of exposure, initial results point towards the fact that patterning perovskite nanocrystals with this type of high-energy radiation is possible and an interesting route to explore in the future. **Figure S1** shows some examples of EUV patterned lines (100 nm pitch) at different doses. From doses of about 475 mJ cm^{-2} we start to see contrast and interrupted lines of $CsPbBr_3$ left on the substrate after development. From 600 mJ cm^{-2} and up we observe well defined lines of nanocrystals. The size dispersion in the nanocrystal size, however, disrupts smooth line edges.

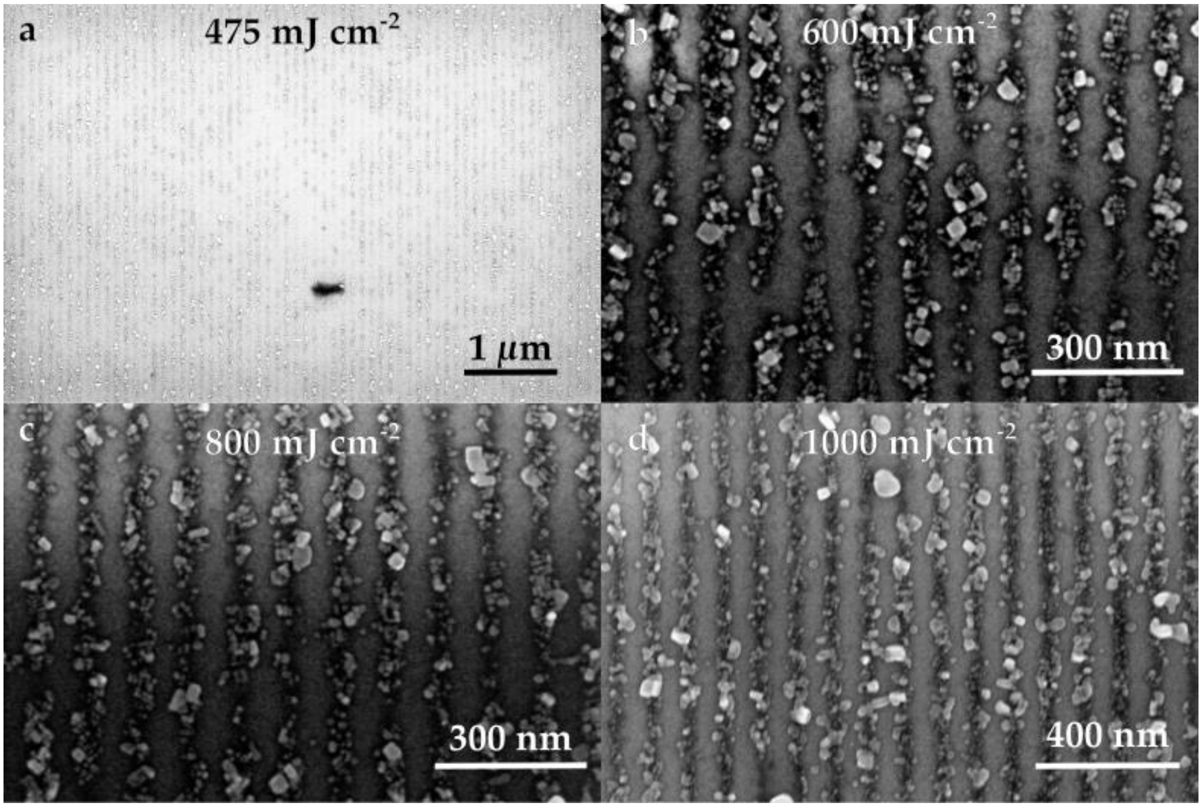

Figure S1. SEM images of EUV exposed and developed perovskite nanocrystal structures. The lines were written with EUV-interference lithography at (a) 475 mJ cm^{-2}, (b) 600 mJ cm^{-2}, (c) 800 mJ cm^{-2} and (d) 1000 mJ cm^{-2}. Samples were developed in a (1:1 %vol) mixture of THF and chloroform.

S2. Overexposed perovskite nanostructures

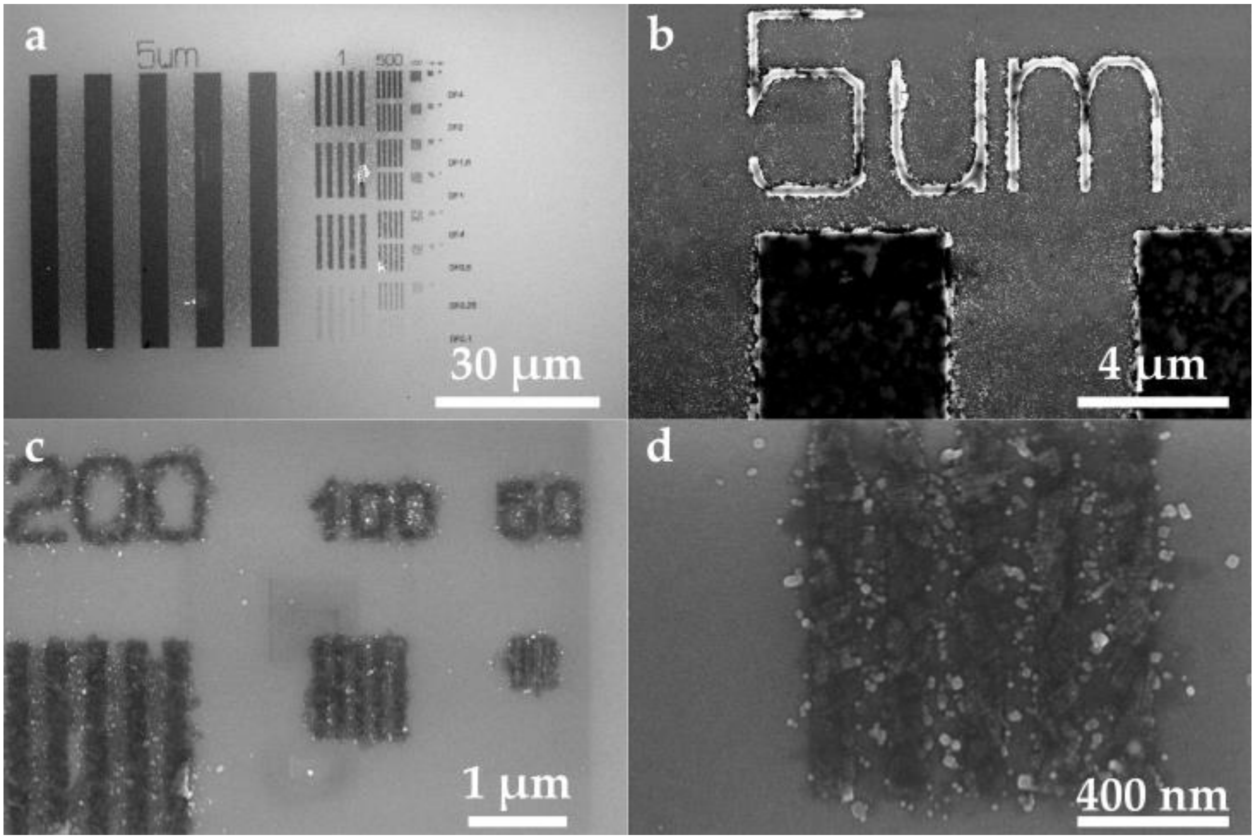

Figure S2. SEM images of overexposed perovskite nanocrystal structures. (a) shows different sized lines (from left to right 5 μm, 1 μm, 500 nm, 200 nm, 100 nm and 50 nm) exposed to different doses (top to bottom 8000, 4000, 3000, 2000, 1500, 1000, 500 and 200 μC cm^{-2}; text is exposed to 10 000 μC cm^{-2}). It is clear that the increasing dose leads to increasing contrast between the substrate and the exposed patterns. However, at higher doses (from 2000, but more clearly from 4000 μC cm^{-2}) there are also more nanocrystals staying behind on the substrate due to overexposure or blurring of the intended patterns. This is an effect of electrons scattering outside of the intended region and causing cross-linking reactions outside the intended area. (b) Zoom-in of the top of 5 μm lines exposed to 2000 μC cm^{-2} and text exposed to 10 000 μC cm^{-2}. (c) Zoom-in of top of 200 nm, 100 nm and 50 nm lines exposed to 8000 μC cm^{-2}. (d) Zoom-in of top of 100 nm exposed to 8000 μC cm^{-2}.

S3. Raw PL spectra $CsPbBr_3$ before and after exposure

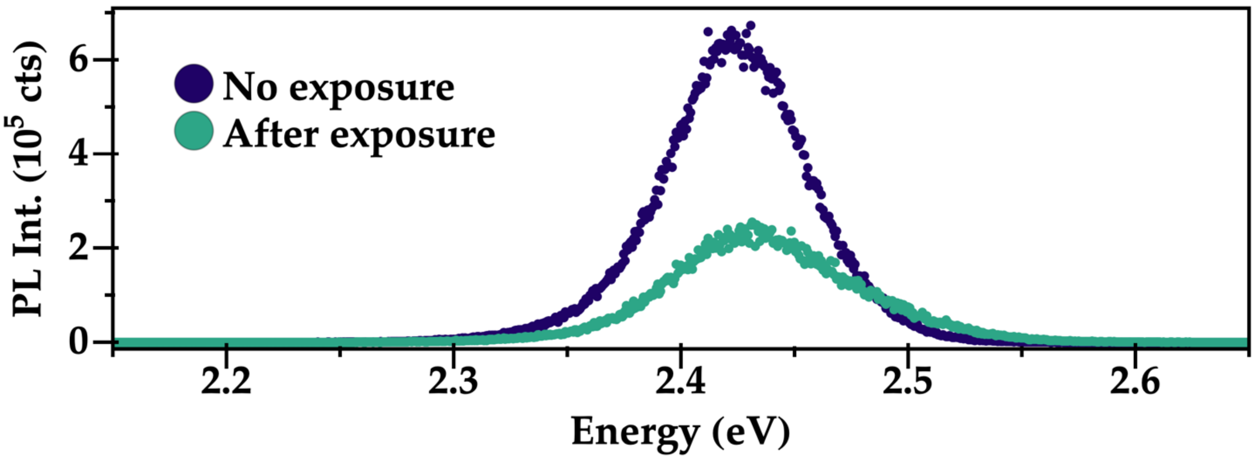

Figure S3. Raw PL spectra of exposed (2000 μC cm^{-2}) and unexposed $CsPbBr_3$**.** After exposure we observe a reduction of PL intensity as well as a broadening on the high energy side. The normalized data can be found in **Figure 3**a of the main text. Data is taken from PL map shown in **Figure 4**c of the main text.

S4. Optical microscope images of e-beam exposed perovskite nanocrystal films

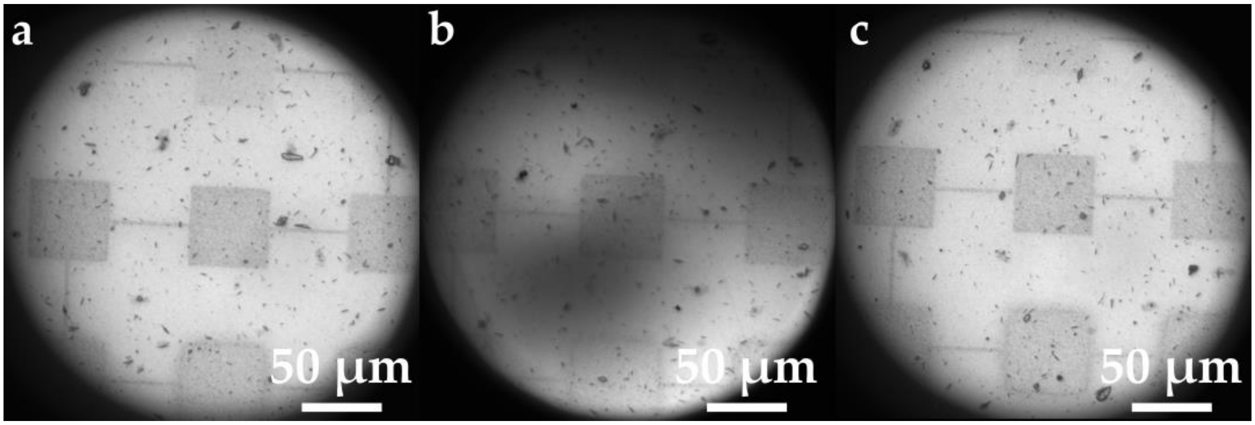

Figure S4. Optical microscope images of 3 different e-beam exposed perovskite nanocrystal films. Exposure to the e-beam alters the absorbance of the films, as also observed in **Figure 4.**

S5. Absorption and reflectance maps of exposed films

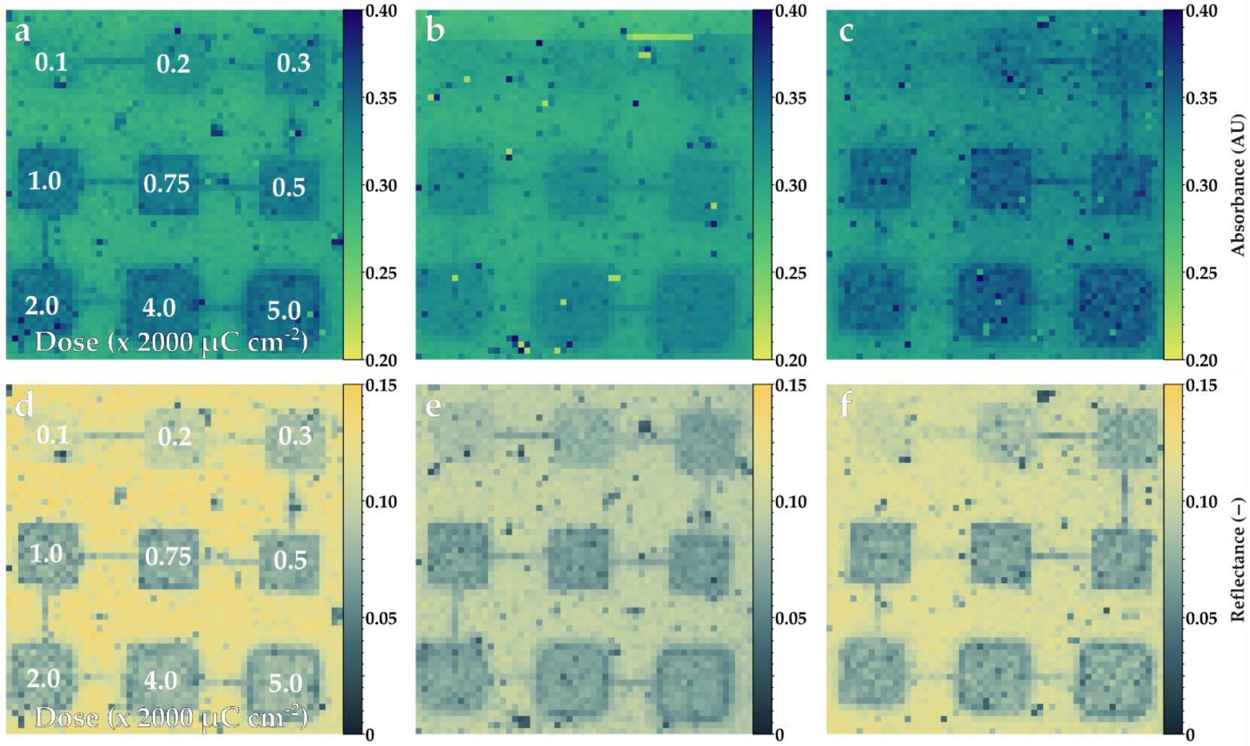

Figure S5. Absorption (a-c) and reflectance (d-f) maps of exposed $CsPbBr_3$ films. Exposure doses are the same for all samples. White labels indicate the dose factor, which should be multiplied with 2000 μC cm^{-2}. Individual exposure fields are 50 μm x 50 μm. Absorption and reflectance was recorded at the same time. Overall summary of these measurements can be found in the main text (**Figure 4b**).

S6. AFM of exposed but undeveloped perovskite films

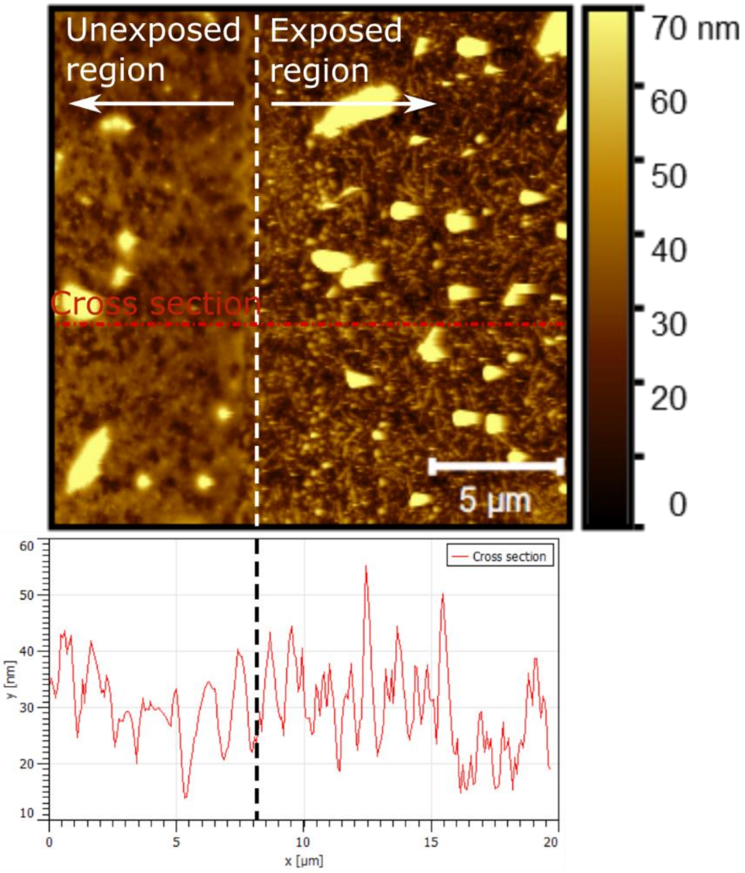

Figure S6. AFM of perovskite nanocrystal film exposed to EUV (92 eV). From previous work we know that EUV induces a similar patterning mechanism to e-beam. The edge of the exposed area is measured here. Although a change in roughness can be observed after exposure, the overall thickness of the film does not change significantly.

S7. Transfer matrix optical modeling

We use the python script provided by the McGehee group[2,3] to calculate how changes in refractive index may affect the measured absorbance and reflectance for our system. We model our system with 4 layers: air, $CsPbBr_3$ (active layer, 95 nm), ITO (250 nm), SiO_2 (175 μm), which is comparable to our NC film and substrate. From literature we find the n = 1.8-1.95 and k = 0.16 – 0.63 in $CsPbBr_3$ NCs[4,5] and thin films[6,7] at λ=405 nm, which is the wavelength at which the measurements were performed. We use $k = 0.165$ and check the range of n between 1.70 and 1.95. **Figure S7** shows the results of the calculations in predicting the reflectance, absorbance and transmission of the modelled stack as function of n. The values we measured before and after exposure, including a margin of error, is marked in all the plots. We find a good agreement between the model and our measurements for the investigated range of n. A reduction of refractive index from 1.9 by around 0.15 might account for the observed changes in absorbance and reflection. Small deviations between the model and the measurements are still possible as it does not account for changes in thickness or surface roughness, which could influence scattering properties of the film.

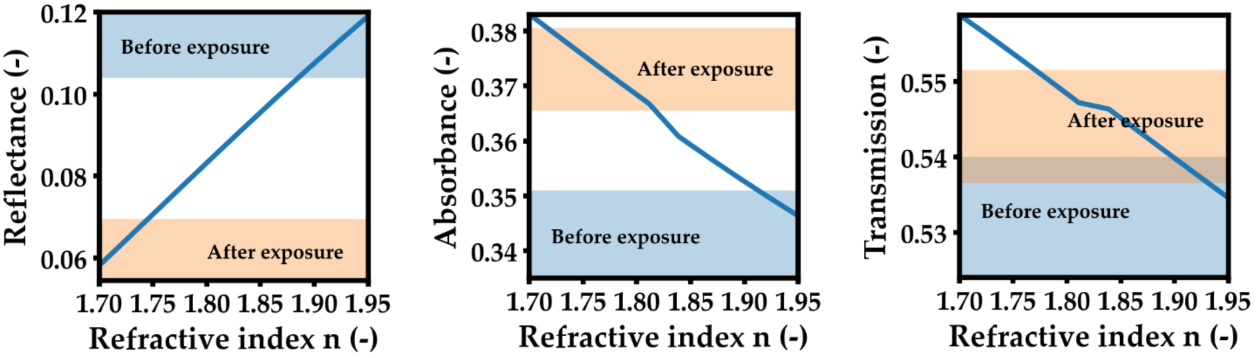

Figure S7. Estimated reflectance, absorbance and transmission of a $CsPbBr_3$ NC layer, as function of the refractive index. A reduction in n may account for the observed changes in our measurements.

S8. TCSPC data of perovskite films

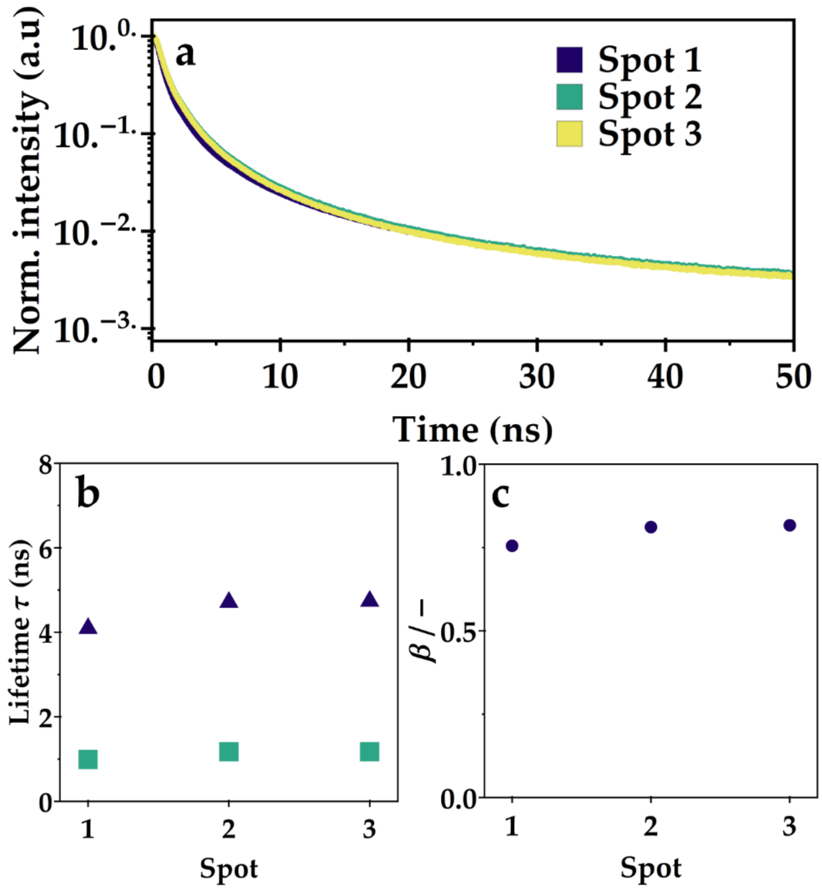

Figure S8. TCPSC data of unexposed perovskite films on different spots (a) and fitted lifetime components (b) τ_1, τ_2 and (c) β.

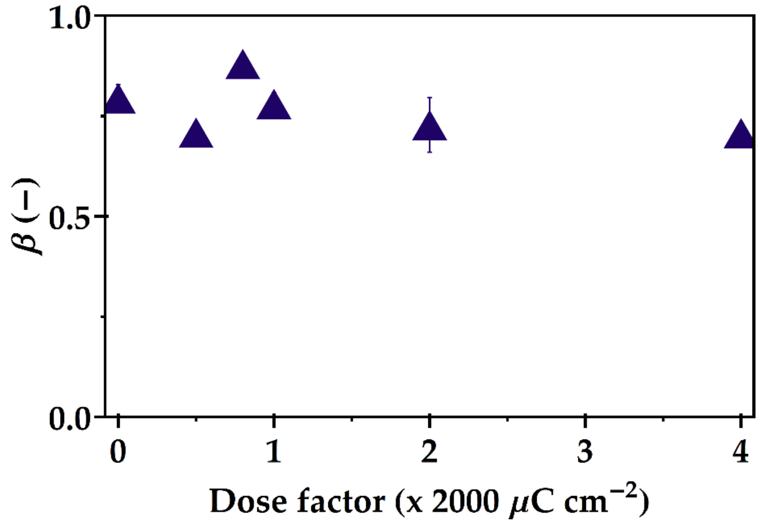

Figure S9. Fitted stretched exponent β of exposed perovskite films.

S9. Monte Carlo simulations of electron trajectories in perovskite film

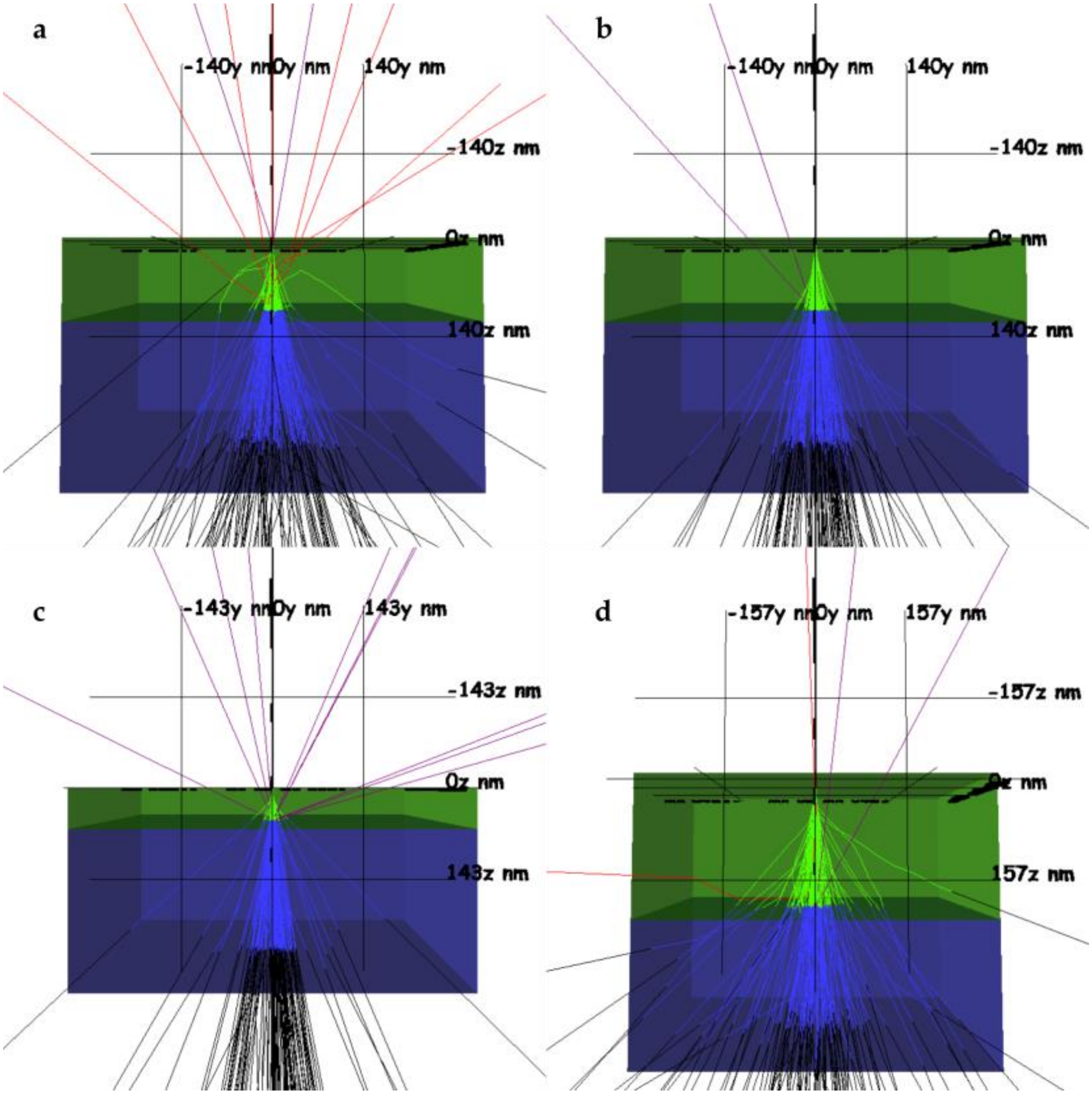

Figure S10. High-energy electron trajectories through films of perovskite. Red traces indicate backscattered electrons, the black traces are leaving the sample and the colored traces indicate the traces through perovskite (green) and silicon (blue). (a) 20 keV electron beam through 100 nm of perovskite, (b) 50 keV electron beam through 100 nm of perovskite, (c) 50 keV electron beam through 50 nm of perovskite, 50 keV electron beam through 200 nm of perovskite. All samples are on top of 200 nm silicon.

We used CASINO 3.3.0.4 software[8] to model the traces of the high-energy electrons through our material in order to show that most electrons will penetrate the film. The film thicknesses we work with are not heavily influencing the lithography process, as is the case with optical lithography, where light is absorbed and significantly less photons reach the substrate compare to the top of the film. For simplicity we modeled the perovskite layer as a bulk $CsPbBr_3$ with a density of 4.33 g cm^{-3}, which is a higher density than nanocrystals would have, since the organic ligands would "dilute" the density, reducing the number of interactions with incoming electrons.

Figure S10 shows the 4 cases we looked at: (i) 20 keV electron beam on 100 nm perovskite, (ii) a 50 keV e-beam on 100 nm perovskite, (iii) a 50 keV e-beam on 50 nm perovskite and (iv) 50 keV e-beam on 200 nm perovskite, all on top of 200 nm silicon. We used a thin layer of Si to more clearly show the trajectories through the perovskite layer. We use the standard Mott model to model 1000 electrons, simulate shot noise and allow for the generation of secondary electrons. From all figures, it is clear that most electrons penetrate the perovskite layer fully. Using 50 keV (**Figure S10**b) electrons reduces the number of backscattered electrons compared to using 20 keV (**Figure S10**a), which should be beneficial for blur and line-edge roughness. Next to this, the beam broadens less when penetrating the material as is clear from the width of the cone leaving the material. When changing the thickness of the perovskite, the behavior of the electrons changes. With a thinner layer of 50 nm (**Figure S10**c) the influence of the substrate becomes more substantial and we again see more backscattering. With a thicker perovskite layer of 200 nm (**Figure S10**d) there is more elastic scattering inside the perovskite film, causing a broadening of the cone of electrons. In both cases this may have a small influence on the blurring, but the vast majority of the electrons still penetrates the film fully in a narrow cone. It should also be noted

that with increasing film thickness, the width of the features that can be written will need to increase as well to reduce the risk of pattern collapse.

References

(1) Solak, H. H. Nanolithography with Coherent Extreme Ultraviolet Light. *Journal of Physics D: Applied Physics* **2006**, *39* (10), R171–R188. https://doi.org/10.1088/0022-3727/39/10/R01.

(2) Burkhard, G. F.; Hoke, E. T. Transfer Matrix Optical Modeling https://web.stanford.edu/group/mcgehee/transfermatrix/TransferMatrix.pdf (accessed 2021 -09 - 03).

(3) Burkhard, G. F.; Hoke, E. T.; McGehee, M. D. Accounting for Interference, Scattering, and Electrode Absorption to Make Accurate Internal Quantum Efficiency Measurements in Organic and Other Thin Solar Cells. *Advanced Materials* **2010**, *22* (30), 3293–3297. https://doi.org/10.1002/ADMA.201000883.

(4) He, S.; Kumar, N.; Beng Lee, H.; Ko, K. J.; Jung, Y. J.; Il Kim, J.; Bae, S.; Lee, J. H.; Kang, J. W. Tailoring the Refractive Index and Surface Defects of CsPbBr3 Quantum Dots via Alkyl Cation-Engineering for Efficient Perovskite Light-Emitting Diodes. *Chemical Engineering Journal* **2021**, *425*, 130678. https://doi.org/10.1016/J.CEJ.2021.130678.

(5) Qaid, S. M. H.; Ghaithan, H. M.; Al-Asbahi, B. A.; Aldwayyan, A. S. Investigation of the Surface Passivation Effect on the Optical Properties of CsPbBr3 Perovskite Quantum Dots. *Surfaces and Interfaces* **2021**, *23*, 100948. https://doi.org/10.1016/J.SURFIN.2021.100948.

(6) Zhao, M.; Shi, Y.; Dai, J.; Lian, J. Ellipsometric Study of the Complex Optical Constants of a CsPbBr3 Perovskite Thin Film. *Journal of Materials Chemistry C* **2018**, *6* (39), 10450–10455. https://doi.org/10.1039/C8TC03222J.

(7) Yan, W.; Mao, L.; Zhao, P.; Mertens, A.; Dottermusch, S.; Hu, H.; Jin, Z.; Richards, B. S. Determination of Complex Optical Constants and Photovoltaic Device Design of All-Inorganic CsPbBr3 Perovskite Thin Films. *Optics Express, Vol. 28, Issue 10, pp. 15706-15717* **2020**, *28* (10), 15706–15717. https://doi.org/10.1364/OE.392246.

(8) Demers, H.; Poirier-Demers, N.; Ré, A.; Couture, A. L.; Joly, D.; Guilmain, M.; de Jonge, N.; Drouin, D. Three-Dimensional Electron Microscopy Simulation with the CASINO Monte Carlo Software. https://doi.org/10.1002/sca.20262.
